# Sustained improvements in students’ mental health literacy with use of a mental health curriculum in Canadian schools

**DOI:** 10.1186/s12888-014-0379-4

**Published:** 2014-12-31

**Authors:** Alan Mcluckie, Stan Kutcher, Yifeng Wei, Cynthia Weaver

**Affiliations:** Faculty of Social Work, University of Calgary, Halifax, Canada; Department of Psychiatry, Dalhousie University and Sun Life Financial Chair in Adolescent Mental Health, Halifax, Canada; Sun Life Financial Chair in Adolescent Mental Health team, Dalhousie University and IWK Health Centre, Halifax, Canada; Adolescent Unit, Ontario Shores Centre for Mental Health Sciences, Whitby, Canada; IWK Health Centre, 5980 University Avenue, NS B3K 6R8 Halifax, NS Canada

**Keywords:** Mental health literacy, Curriculum-based intervention, Youth mental health, Stigma reduction

## Abstract

**Background:**

Enhancement of mental health literacy for youth is a focus of increasing interest for mental health professionals and educators alike. Schools are an ideal site for addressing mental health literacy in young people. Currently, there is limited evidence regarding the impact of curriculum-based interventions within high school settings. We examined the effect of a high-school mental health curriculum (*The Guide*) in enhancing mental health literacy in Canadian schools.

**Methods:**

We conducted a secondary analysis on surveys of students who participated in a classroom mental health course taught by their usual teachers. Evaluation of students’ mental health literacy (knowledge/attitudes) was completed before and after classroom implementation and at 2-month follow-up. We used paired-samples t-tests and Cohen’s *d* value to determine the significance and impact of change.

**Results:**

There were 265 students who completed all surveys. Students’ knowledge significantly improved between pre- and post-tests (*p* < 0.001; *d* = 0.90) and was maintained at follow-up (*p* < 0.001; *d* = 0.73). Similarly, attitude significantly improved between pre- and post-tests (*p* < 0.001; *d* = 0.25) and was significantly higher at follow-up than base-line (*p* < 0.007; *d* = 0.18)

**Conclusions:**

*The Guide*, applied by usual teachers in usual classroom curriculum, may help improve student knowledge and attitudes regarding mental health. This is the first study to demonstrate the positive impact of a curriculum-based mental health literacy program in a Canadian high school population.

**Electronic supplementary material:**

The online version of this article (doi:10.1186/s12888-014-0379-4) contains supplementary material, which is available to authorized users.

## Background

Mental health and mental disorders in youth are a major public health concern. Approximately one in five young Canadians may experience a mental disorder requiring professional care [1-5]. The majority onset prior to 25 years of age and often follow an extended pattern of remission and relapse over the life span [1]. Despite the great burden of disability they create [5,6], the majority of youth needing care do not receive it [7-10]. Unrecognized and/or untreated mental disorders can lead to a variety of negative outcomes including poor educational/vocational achievement, problematic interpersonal and family functioning, and reduced life expectancy due to associated medical conditions and suicide [11,12]. Interventions leading to early identification along with effective treatment increase the possibility of positive outcomes [5, 13].

Increasingly, policy makers, mental health professionals and educators are recognizing the important role that schools may play in addressing the mental health needs of young people [13-20]. For example, the Department for Education in the United Kingdom has recently released a seminal policy on this issue [18]. Earlier, the U.S. Surgeon General’s report has identified schools as important in early identification and treatment of mental disorders [21]. The World Health Organization (WHO) advocates for mental health promotion/prevention in schools [22]. School-based mental health interventions are endorsed by Canadian mental health policy documents, such as the *Evergreen Framework: a Child and Adolescent Mental Health Framework for Canada* [14], Ontario’s Policy Framework for Child and Youth Mental Health [23], *Nova Scotia’s Kids and Learning First Plan* [24] and non-government organizations such as Children’s Mental Health Ontario [25].

Essential to enhancement of both individual and population health is the improvement of health literacy [26,27] and this necessity has been endorsed by international health organizations such as the World Health Organization [28] and nationally by provinces [29] and federal organizations [30] alike. Similarly, mental health literacy is an essential and foundational component for mental health promotion, prevention, early identification and intervention. Mental health literacy has been defined as “knowledge and beliefs about mental disorders which aid their recognition, management or prevention” [31]. More recently this definition has been enhanced to be: the capacity to understand how to enhance and maintain good mental health; understand mental disorders and their treatments; decrease stigma against those living with mental disorder; and enhance help-seeking efficacy [32]. This definition is consistent with global public health frameworks such as the Ottawa Charter of Health Promotion [33] and a comprehensive approach to addressing mental health as promoted by national [14] and international [34] mental health frameworks.

Schools are an ideal venue in which to embed mental health literacy at both the individual and population levels because schools are where most young people can be reached and classroom-based educational activities are familiar to students and educators alike [19,35]. Embedding mental health literacy into existing school curriculum can potentially reach all students, normalizes mental health as part of everyday activities and engages teachers to become literate in youth mental health as part of their professional activities [19,36].

Current school-based mental health literacy programs have usually followed one of two directions: programs addressing specific conditions (e.g., depression, eating disorders, and schizophrenia) [37] and programs for general understanding of mental health and mental disorders [38]. A systematic review, conducted by Wei and colleagues [32] identified 15 reviews that examined the outcomes of high school-based general mental health literacy programs. The majority of these were found to be associated with no or only minor gains in the students’ knowledge and/or attitudes towards mental disorders. This review revealed that only one study [39] has reported on a mental health literacy intervention in Canadian high school students [32].

We examined the impact of a recently developed school-based mental health literacy resource, Mental Health & High School Curriculum Guide (*The Guide*) [40] on students’ knowledge and attitudes related to mental health. *The Guide* is a mental health literacy resource designed to inform junior high and high school curriculum. It was developed in collaboration between mental health experts, educators and the Canadian Mental Health Association (CMHA), a national mental health non-governmental organization. *The Guide* has been endorsed by the *Canadian Association for School Health,* is certified by *Curriculum Services Canada*, is embedded into the Provincial grade nine school curriculum in Nova Scotia, is currently being used in hundreds of additional schools across Canada and was extensively field-tested, in both English and French schools prior to its application in this study [41].

*The Guide* is a manualized mental health literacy resource consisting of six modules that are delivered in 10–12 hours of class time through a mix of didactic instruction, group discussion, group activities, self-directed learning and video presentations. Modules cover topics including stigma and mental illness, understanding mental health and mental illness, information on specific mental illness, first voice experiences and impact of mental illnesses on individuals and families, help-seeking and the importance of positive mental health. *The Guide* also includes a teacher self-study module that provides teachers more in-depth understanding of the materials they teach. It can be obtained at www.teenmentalhealth.org. In usual application, teachers using *The Guide* in their classrooms undergo a single training day to familiarize themselves with the materials. The effect of this on teachers own mental health literacy has been examined and has shown significant and substantial positive impact on their knowledge and attitudes [42].

This study extends evaluation of the use of the mental health literacy curriculum delivered by usual classroom teachers in usual school function. It examines the impact of *The Guide* on the mental health literacy of high school students. Specifically, we were interested in evaluating if students’ knowledge and attitudes related to mental health changed following participation in *The Guide* mental health literacy curriculum delivered by their usual classroom teachers, as part of their usual school curriculum in their usual classroom settings.

## Methods

Between February and June 2012 *The Guide* was implemented as a regular component of Grade 9 health classes in high schools of the four school boards in the regions of Durham and Peterborough/Kawartha (i.e., Durham District School Board [DDSB], Durham Catholic District School Board [DCDSB], Kawartha Pine Ridge School Board [KPRSB], and Peterborough Victoria Northumberland and Clarington Catholic District School Board [PVNCCDSB]), in the province of Ontario, Canada. Durham region is an administrative area, just east of the largest city in Canada (Toronto). Its population is predominantly urban, of European ethnic origin with immigrants from the Caribbean and Asia accounting for most of the remainder. About 14% of its population is between the ages of 15 – 24 years, over eighty percent of families are two parent families and the average household income is greater than the Ontario average [38]. Schools were representative of the region.

For evaluation purposes, school personnel collected anonymous survey materials from students regarding knowledge and attitudes towards mental health before and after the delivery of *The Guide* (pre-test and post-test) as well as at 2-month follow-up.

A secondary analysis was conducted on this anonymous survey material originally gathered for program evaluation purposes. We examined available student surveys and compared responses gathered before the implementation of *The Guide* (pre-test), with those gathered immediately following the implementation of *The Guide (*post-test) and also with responses gathered at 2-month follow-up. Ethical approval was obtained from the Research Ethics Board of OSCMHS, affiliated with the University of Toronto along with the research committees of each participating school board.

### Measures

The knowledge and attitude survey is composed of two sections. The first includes 28 items assessing general mental health knowledge, corresponding to materials contained in the six modules of *The Guide*. Items are presented in a True/False and “Do Not Know” format. The Cronbach’s alpha for internal consistency is 0.71. The second section uses eight items to examine attitudes related to mental disorders/illness. These items include statements about mental disorders and/or individuals with a mental illness and ask respondents to select their level of agreement using a seven point Likert scale (i.e., from “strongly disagree” to “strongly agree”) (see Additional file 1 for the student survey). To ensure the anonymity of the survey, five linking questions were predesigned to match participants’ pre-test, post-test and follow-up test. This includes participants completing the survey sheet with their first pet name, birth month, postal codes, shoe size, and the last two digits/number of their phone number. Unmatched surveys were excluded from the data analysis.

### Statistical Analysis

In order to determine the significance of observed scores between pre-test, post-test and follow-up, a series of paired-samples *t-*tests were employed to compare changes in the total number of correct scores on survey items pertaining to general mental health knowledge and total attitude scores. A Bonferroni correction was applied in order to reduce the chance of obtaining false-positive results (i.e., Type I errors) associated with the use of multiple *t-*tests. Therefore, significance levels α = 0.05 were adjusted to significance level of 0.00834 (α of .05/6 *t* tests). SPSS was used to conduct the analysis.

## Results

The total sample size was 409 (269 female and 140 males) for pre- and post-assessments. Of this sample, 265 participants were available for follow-up analysis.

Prior to their exposure to *The Guide*, students responded correctly to 15 of 28 questions (53%) pertaining to general mental health. Following exposure to *The Guide*, correct responses improved to 18 of 28 (64%), an average which was maintained at the 2-month follow-up period. Student knowledge scores following exposure to *The Guide* (*M* = 18.55*, SD* = 4.18) is significantly and substantively higher than baseline student knowledge scores (*M* = 14.91, *SD* = 4.02), *t*(408) = 18.22, *p <* 0.001, *d* = 0.90). Students’ knowledge scores at the 2-month follow-up period (n = 265) were also significantly higher (*M* = 18.22*, SD* = 4.20) compared to base-line (pre-test) knowledge scores (*M* = 15.23*, SD* = 4.04), *t*(264) = 11.92, *p <* 0.001, *d* = 0.73). Refer to Table 1 for the results of the paired-samples *t* tests comparing students’ changes in mental health knowledge between pre-test, post-test and follow-up and *d* statistics for the effect size of the training impact.

**Table 1:** **Change in knowledge scores from pre-test, post-test, and follow-up**

**Pre-test**	**Post-test**	**Follow-up**	***t test comparison***	***d statistic***
14.91(4.02)	18.55(4.18)		*t*(408) = 18.22, *p* < 0.001	0.90
15.23(4.04)		18.22(4.20)	*t*(264) = 11.92, *p* < 0.001	0.73

*Note*. The *d* statistics represent the effect size of the training, describing the magnitude of difference between the pre-test and post-test or between the pre-test and follow-up.

The mental health attitudes assessment allows for scores ranging from 8 to 56, where larger scores are indicative of more positive attitudes. Following exposure to *The Guide*, students showed significant but numerically small improvements in attitude between pretest surveys with a modest effect size (*M* = 34.09, *SD* = 5.48) and post-test surveys (*M* = 35.34, *SD* = 5.82), *t*(347) = 4.78, *p <* 0.001, *d* = 0.25. Of the sample, 251 students also completed the 2-month follow-up survey. While the 2-month follow up attitude scores decreased somewhat from the immediate post intervention scores, they still remained higher than baseline and the effect size was now smaller (*M* = 33.34, *SD* = 4.24, pre-test; M = 34.26, SD = 4.10, 2-month follow-up), *t*(233) = 2.73, *p <* 0.007, *d* = 0.18). Further, there were no significant differences identified between post-test and 2 month follow-up (p > 0.05), suggesting attitudes maintained overtime. Differences between pre-test and post-test attitude scores remained after the Bonferroni correction was applied, but as the sample size in the follow-up group was smaller and not identical to that that of the immediate post survey group the baseline attitudes score appeared somewhat lower than that in the larger sample. Refer to Table 2 for the results of the paired-samples *t*-tests comparing students’ changes in mental health attitude between pre-test, post-test and follow-up and *d* statistics for the effect size of the training impact.

**Table 2:** **Change in Students’ attitudes scores from pre-test, post-test, and follow-up**

**Pre-test**	**Post-test**	**Follow-up**	***t test comparison***	***d statistic***
34.09(5.8)	35.34(5.5)		*t*(347) = 4.78, *p* = 0.001	0.25
33.34(4.24)		34.26(4.10)	*t*(233) = 2.73, *p* = 0.007	0.18

*Note*. The *d* statistics represent the effect size of the training, describing the magnitude of difference between the pre-test and post-test or between the pre-test and follow-up.

## Discussion

This study demonstrates that junior/high school mental health curriculum based on *The Guide* may play an important role in significantly, substantively and sustainably improving student knowledge regarding mental health and attitudes towards mental disorders/illness when integrated into usual school curriculum and taught by classroom teachers. To our knowledge this is the first study to demonstrate the significantly positive and sustained impact of a curriculum-based mental health literacy program in a Canadian junior/high school population. Findings from this study show that it is possible to enhance students’ mental health literacy by implementing a mental health curriculum delivered by usual classroom teachers embedded within usual school curriculum based on *The Guide,* therefore setting the foundation for youth mental health promotion, prevention and early identification, and continuing care. This positive outcome does not require the implementation of external “stand alone” mental health interventions, is delivered using existing classroom teachers using well-established pedagogic techniques and avoids the sensationalization of mental health by integration within existing school curriculum.

These findings related to change in student knowledge and attitude are consistent with previous reports demonstrating the positive impact of teacher training on the use of *The Guide* related to significant and substantial changes in mental health knowledge and decrease in stigma amongst teachers [42]. Taken as a whole, these data suggest that providing teachers with the resources and training to implement mental health literacy into usual school curriculum and then using *The Guide* to direct classroom application of this material can improve knowledge and attitudes of both teachers and students alike.

These findings further support the perspective of authors [43,44,45] who argue that adolescents’ negative attitudes towards individuals with mental disorders may be amenable to change through educational programming related to mental illness and that this may lead to improved attitudes and increased access of mental health care by youth [46,47]. This approach, applied in this study, differs markedly from commonly employed stand-alone stigma reduction programs, which have not been extensively shown to decrease stigmatizing attitudes in adolescents [48,49]. In this study, the use of a school-based curriculum delivered by usual classroom teachers based on *The Guide,* demonstrated significant reduction in stigmatizing attitudes following implementation, and although attitude scores decreased somewhat over time, they still remained higher than at baseline. This suggests that embedding mental health literacy into usual school curriculum may be an effective and sustained anti-stigma approach for young people.

Educators, health professionals and policy makers alike have recognized the importance of mental health literacy [19,31,50]. For example, a recent Canadian survey on educators’ perspectives of school mental health demonstrated that they considered mental health to be extremely important, but lacked confidence in addressing it due to a lack of knowledge [51].

Similar to health literacy, mental health literacy is a necessary foundation for mental health promotion, prevention and interventions [52,53]. Therefore, exposure of young people to effective mental health literacy programs can be considered a fundamental first step in addressing a host of mental health related activities. Unfortunately, prior to the results reported herein, most school-based mental health literacy approaches have not demonstrated robust or sustained outcomes in knowledge and attitudes in students [32]. Some researchers [54] have argued that this may be due to the type of material provided and method by which it is provided, often in stand-alone extra-curricular workshop sessions. Integration of mental health literacy materials in usual junior/high school curriculum taught by trained teachers using *The Guide* may mitigate these concerns.

This study examined changes in knowledge and attitudes through the implementation of *The Guide*. Its positive results encourage further research, particularly into whether this approach contributes to positive behavioral change (such as increased help-seeking behaviors, decreased rates of bullying) in both students and teachers who are exposed to *The Guide*-directed curriculum. Such studies are currently being planned. Further studies, evaluating teachers reports on the challenges and impact of implementing *The Guide* resource in their classrooms are underway.

This study has some limitations. It is a secondary cross-sectional survey; the time to the second point of post-exposure was limited to two months and did not allow for a control group or randomization. Further, the slightly high attrition (35%) at 2-month follow-up requires caution in interpreting the data from this time point. The improvement in attitudes although statistically robust was based on a small absolute score improvement, which raises a question about how much improvement in attitude scale scores may be needed to translate into de-stigmatized behaviors. This problem of interpreting changes in attitudes scores is not unique to this study and future research will need to consider how to determine the relationship between significant changes in attitude scale scores and meaningful changes in attitudes and behavior. Costs of the application were not addressed, but were minimal and the approach lends itself to the creation of teacher training teams that may provide sustainable and cost-effective capacity that is administratively easy to implement. We are aware of these limitations and a randomized control trial and further evaluations that may effectively address them are currently underway.

## Conclusions

This study demonstrates that the application of a curriculum embedded mental health literacy resource (*The Guide),* delivered by usual classroom teachers may play a substantive positive role in facilitating significant and sustained improvements in mental health knowledge and decreases in stigmatizing attitudes in high school students. To our knowledge this is the first study of its kind to demonstrate this degree of robust findings in school-based approaches to mental health literacy among the student population. This approach fits very well with the usual operation of schools, in which usual classroom teachers provide instruction based on defined curriculum. Such an approach does not require significant amounts of external resources added to schools and is not dependent upon mental health experts, which may not be easily available in most jurisdictions. As such, it fits well into whole school approaches and builds on existing professional pedagogic capabilities of teachers, while at the same time “normalizing” the approach to mental health as not a stand-alone item, but as integrated into usual learning and school activity. In this way, it may have global applicability as world-wide, common elements of schools include the presence of curriculum and the use of teachers to deliver it. Indeed, international applications of this approach are now being evaluated.

## Additional file

Additional file 1:
**Student Mental Health Literacy Survey.**

## References

[1] Kessler RC, Berglund P, Demler O, Jin R, Merikangas KR (2005). Lifetime prevalence and age of-onset distributions of DSM-IV disorders in the national comorbidity survey replication. Arch Gen Psychiatry.

[2] Offord DR, Boyle MH, Szatmari P, Rae-Grant NI, Links PS, Cadman DT, Byles JA, Crawford JW, Munroe Blum H, Byrne C, Thomas H, Woodward CA (1987). Ontario child health study: Six-month prevalence of disorder and rates of service utilization. Arch Gen Psychiatry.

[3] Offord DR, Boyle MH, Fleming JE, Munroe Blum H, Rae Grant NI (1989). Ontario health study: summary of selected results. Can J Psychiatry.

[4] Kirby MJL, Keon WJ (2004). Mental Health, Mental Illness and Addiction: Overview of Policies and Programs in Canada.

[5] Waddell C, Shepherd C (2002). Prevalence of Mental Disorders in Children and Youth: A Research Update Prepared for the British Columbia Ministry of Children and Family Development.

[6] World Health Organization (2001). The World Health Report. Mental Health: new Understanding, new Hope.

[7] Burns BJ, Costello EJ, Angold A, Tweed DL, Stangl DK, Farmer EM, Erkanli A (1995). Children’s mental health service use across service sectors. Health Aff.

[8] Foster S, Rollefson M, Doksum T, Noonan D, Robinson G, Teich J*: School mental health services in the United States, 2002–2003*. Rockville, MD: Center for Mental Health Services, Substance Abuse and Mental Health Services Administration; 2005 (DHHS Pub. No. (SMA) 05–4068).

[9] Leaf RJ, Alegria M, Cohen R, Goodman SH, Horowtiz SM, Hoven CW, Narrow WE, Vaden-Kiernan M, Regier DA (1996). Mental Health service use in the community and schools: results from the four-community MECA study. J Am Acad Child Adolesc Psychiatry.

[10] Waddell C, Offord DR, Shepherd CA, Hua JM, McEwan K (2002). Child psychiatric epidemiology and Canadian public policy-making: the state of the science and the art of the possible. Can J Psychiatry.

[11] Bhatia S (2007). Childhood and adolescent depression. Am Fam Physician.

[12] Kessler RC, Foster CL, Saunders WB, Stang PE (1995). Social consequences of psychiatric disorders I: educational attainment. Am J Psychiatry.

[13] Kutcher S (2011). Facing the challenge of care for child and youth mental health in Canada: a critical commentary, five suggestions for change and a call to action. Healthc Q.

[14] Kutcher S, McLuckie A (2010). Evergreen: A Child and Youth Mental Health Framework for Canada.

[15] McLaughlin MJ, Leone PE, Meisel S, Henderson K (1997). Strengthen school and community capacity. J Emot Behav Disord.

[16] Kieling C, Baker-Henningham H, Belfer M, Conti G, Ertem I, Omigbodun O, Rohde LA, Srinath S, Ulkuer N, Rahman A (2011). Child and adolescent mental health worldwide: evidence for action. Lancet.

[17] McGorry PD, Purcell R, Goldstone S, Amminger GP (2011). Age of onset and timing of treatment for mental and substance use disorders: implications for preventive intervention strategies and models of care. Curr Opin Psychiatry.

[18] Department for Education, UK: **Mental Health and Behaviour in Schools** [https://www.gov.uk/government/uploads/system/uploads/attachment_data/file/326551/Mental_Health_and_Behaviour_-_Information_and_Tools_for_Schools_final_website__2__25-06-14.pdf]

[19] Atkins M, Hoagwood K, Kutash K, Seidman E (2010). Toward the integration of education and mental health in schools. Adm Policy Ment Health.

[20] Rowling L, Kutcher S, Wei Y, Weist M (2015). Developing and Sustaining Mental Health and Wellbeing in Australian Schools. International School Mental Health for Adolescents: Global Opportunities and Challenges.

[21] U.S. Department of Health and Human Services (1999). Mental Health: A Report of the Surgeon General.

[22] WHO Regional Office for the Western Pacific (1996). Regional Guidelines Development of Health Promoting Schools: A Framework for Action.

[23] Ontario Ministry of Children and Youth Services (2006). A Shared Responsibility: Ontario’s Policy Framework for Child and Youth Mental Health.

[24] Nova Scotia Government: *Kids and learning first: A plan to help every student succeed.* NS. Government of Nova Scotia; 2012 [http://novascotia.ca/kidsandlearning/pub/KL-en.pdf] Accessed 7 Jan 2015.

[25] **Children’s Mental Health Ontario** [http://www.kidsmentalhealth.ca/]

[26] Kickbusch IS (2001). Health literacy: addressing the health and education divide. Health Promot Int.

[27] Nielsen-Bohlman L, Panzer AM, Kindig DA, Committee on Health Literacy (2004). *Health literacy: A prescription to avoid confusion*.

[28] Kickbusch I, Pelikan JM, Apfel F, Tsouros A (2013). Health Literacy: The Solid Facts.

[29] Mitic W, Rootman I (2012). Approach for Improving Health Literacy for Canadians.

[30] Public Health Agency of Canada: *Canadian perinatal health report, 2008 edition.* Health Canada; 2008.

[31] Jorm AF, Korten AE, Jacomb PA, Christensen H, Rodgers B, Pollitt P (1997). ‘Mental health literacy’: a survey of the public’s ability to recognize mental disorders and their beliefs about the effectiveness of treatment. Med J of Aust.

[32] Wei Y, Hayden J, Kutcher S, Zygmut A, McGrath P (2013). The effectiveness of school-based mental health literacy programs to enhance knowledge, change attitudes and improve help-seeking behaviours in youth: a systematic review. Early Interv Psychiatry.

[33] World Health Organization (1986). Ottawa charter for health promotion: first international conference on health promotion. Can J Public Health.

[34] World Health Organization (2012). Comprehensive Mental Health Action Plan, 2013–2020.

[35] Wyn J, Cahill H, Holdsworth R, Rowling L, Carson S (2000). MindMatters, a whole-school approach promoting mental health and wellbeing. Aust NZ J of Psychiat.

[36] Ringeisen H, Henderson K, Hoagwood K (2003). Context matters: Schools and the “research to practice gap” in children’s mental health. School Psychology Rev.

[37] Merritt RK, Price JR, Mollison J, Geddes JR (2007). A cluster randomized controlled trial to assess the effectiveness of an intervention to educate students about depression. Psychol Med.

[38] Durham Region Planning Department (2009). Durham Region Profile Demographics and Socio-Economic Data.

[39] Pinfold V, Stuart H, Thornicroft G, Arboleda-Florez J (2005). Working with young people: the impact of mental health awareness programs in schools in the UK and Canada. World Psychiatry.

[40] Kutcher S, The Canadian Mental Health Association (2009). The mental health & high school curriculum: Understanding mental health and mental illness.

[41] Kutcher S, Wei Y (2013). Challenges and solutions in the implementation of the school-based pathway to care model: the lessons from Nova Scotia and beyond. Can J Sch Psychology.

[42] Kutcher S, Wei Y, McLuckie A, Bullock L (2013). Educator mental health literacy: a programme evaluation of the teacher training education on the mental health & high school curriculum guide. Adv Sch Ment Health Promot.

[43] Ng P, Chan KF (2002). Attitudes towards people with mental illness: Effects of a training program for secondary school students. Int J Adolesc Med Health.

[44] Corrigan PW, Lurie BD, Goldman HH, Slopen N, Medasani K, Phelan S (2005). How adolescents perceive the stigma of mental illness and alcohol abuse. Psychiatr Serv.

[45] Leavey J (2005). Youth experiences of living with mental health problems: emergence, loss, adaption and recovery. Can J Comm Ment Health.

[46] Boldero J, Fallon B (1995). Adolescent help-seeking: what do they get help for and from whom?. J Adolesc.

[47] Moses T (2009). Self**-**labeling and its effects among adolescents diagnosed with mental disorders. Soc Sci Med.

[48] Dalky HF (2012). Mental illness stigma reduction interventions: review of intervention trials. West J Nurs Res.

[49] Stuart H, Arboleda-Florez J, Sartorius N: *Paradigms Lost: Fighting Stigma and the Lessons Learned*. New York, Oxford; 2012

[50] Wei Y, Kutcher S, Szumilas M (2011). Comprehensive school mental health, an integrated “School-Based Pathway to Care” model for Canadian secondary schools. McGill J Educ.

[51] School-Based Mental Health and Substance Abuse Consortium Knowledge Translation and Review Team (2012). Survey on School-Based Mental Health and Addictions Services in Canada.

[52] Parker RM, Williams MV, Weiss BD, Baker DW, Davis TC, Doak CC, Doak LG, Hein K, Meade CD, Nurss J, Schwartzberg JG, Somer SA, Davis RM, Riggs JA, Field H, Champion HC, Howe JP, Altman RD, Deitchman SD, Genel M, Karlan MS, Khaleem Khan M, Nielsen NH, Williams MA, Young DC, Schwartzberg J, Bresolin LB, Dickinson BD (1999). Health literacy: report of the council on scientific affairs. JAMA.

[53] Sanders LM, Shaw JS, Guez G, Baur C, Rudd R (2009). Health literacy and child health promotion: implications for research, clinical care, and public policy. Pediatrics.

[54] Begoray DL, Wharf-Higgins J, MacDonald M (2009). High school health curriculum and health literacy: Canadian student voices. Glob Health Promot.

