# Supplementary material for: Sustained improvements in students’ mental health literacy with use of a mental health curriculum in Canadian schools
Source: BMC Psychiatry. 2014 Dec 31;14:379. doi: 10.1186/s12888-014-0379-4 (PMC4300054; doi:10.1186/s12888-014-0379-4)

# Additional file 1

# A

# School Mental Health & *The* *Mental Health & High School Curriculum Guide*

This survey is designed to assess the baseline knowledge regarding school mental health and the Mental Health & High School Curriculum Guide.

What is the name of your school: _________________________.

I identify myself as Male Female

What Grade are you currently in: 9 10 11 12 Other_________________________

What English class are you enrolled in at school: Applied Academic Other

**---------------------------------------------------------------------------------------------------------------------**

To help us match your anonymous responses between surveys done at the start and end of the course please answer **at least** **3 of these 5 questions**. These answers allow you to remain anonymous and still allow us to see if your scores on the survey change before and after participating in the class.

a) The name of your first pet __________________________________________________________________,

b) Your birth **month** _____________, c) Your postal code______________, d) Your shoe size ______________,

e) The last two digits/numbers of your home phone number __________________.

**--------------------------------------------------------------------------------------------------------------------------------------------------**

**Section A:** For each of the following statements select True, False**,** or Do Not Know by marking an **X** in the appropriate box.

| **Question** | **True** | **False** | **Do Not Know** |
| --- | --- | --- | --- |
| 1. Mental health and mental illness both involve the brain and how it functions. |  |  |  |
| 1. People who have mental illness can at the same time have mental health. |  |  |  |
| 1. The brain can affect the way the body functions but the body can not affect the way the brain functions. |  |  |  |
| 1. The frontal lobes of a young person’s brain continue to grow and develop until about the age of 25 years. |  |  |  |
| 1. Three of the functions of the brain include thinking, signaling and behavior. |  |  |  |
| 1. Every person’s mood can fluctuate up and down naturally. |  |  |  |
| 1. The brain acts to help control the functioning of the heart, lungs, and fingers. |  |  |  |
| 1. Both genetic problems and infections can cause the brain to get sick and stop functioning normally. |  |  |  |
| 1. The symptoms of mental illness are caused by abnormal functioning of the brain. |  |  |  |
| 1. People who have a mental illness are frequently violent. |  |  |  |
| **Question** | **True** | **False** | **Do not know** |
| 1. Most people who have a mental illness get well and stay well with treatment. |  |  |  |
| 1. People who have schizophrenia often get a split personality. |  |  |  |
| 1. Vitamins and meditation are good treatments for most mental illnesses. |  |  |  |
| 1. Depression and Bipolar Disorder are two examples of the type of mental illnesses called mood disorders. |  |  |  |
| 1. An anxiety disorder happens when a person’s brain detects the presence of danger – such as a dog attacking. |  |  |  |
| 1. Panic Disorder commonly begins in adolescence. |  |  |  |
| 1. A panic attack comes on suddenly and typically lasts one or more days. |  |  |  |
| 1. Attention Deficit Hyperactivity Disorder has three components including attention problems, hyperactivity, and depression. |  |  |  |
| 1. Suicide in young people is mostly related to bullying and has little to do with mental illness. |  |  |  |
| 1. Self-harming behaviors may sometimes accidentally lead to death. |  |  |  |
| 1. Treatment of mental disorders has three purposes including, relieving symptoms, restoring functioning, and promoting recovery. |  |  |  |
| 1. People with Social Anxiety Disorder experience irrational and excessive fear that they will act in a way that will be humiliating or embarrassing. |  |  |  |
| 1. Anorexia Nervosa is an eating disorder that can lead to death. |  |  |  |
| 1. One important job of the brain is to help the person adapt to the environment. |  |  |  |
| 1. Mental disorders usually begin because of the stresses of everyday life. |  |  |  |
| 1. Psychosis is a disturbance in thinking and perception leading to loss of contact with reality. |  |  |  |
| 1. The main symptoms of Schizophrenia are delusions and hallucinations. |  |  |  |
| 1. Medicines should be used to treat all mental disorders. |  |  |  |

**Section B:** This section of the survey is designed to find out about your attitudes toward the statement.

For each of the following statements please mark an **X** in the box that you feel best describes your attitude toward the statement. Please select only one answer for each statement.

|  | **Strongly Disagree** | **Disagree** | **Disagree a little** | **Not sure** | **Agree a little** | **Agree** | **Strongly**  **Agree** |
| --- | --- | --- | --- | --- | --- | --- | --- |
| 1. It is easy to tell when someone has a mental illness because they usually act in a strange or bizarre way |  |  |  |  |  |  |  |
| 2. A mentally ill person should not be able to vote in an election |  |  |  |  |  |  |  |
| 3. Most people who have a mental illness are dangerous and violent |  |  |  |  |  |  |  |
| 4. Most people with a mental illness can have a good job and a successful and fulfilling life |  |  |  |  |  |  |  |
| 5. I would be willing to have a person with a mental illness at my school |  |  |  |  |  |  |  |
| 6. I would be happy to have a person with a mental illness become a close friend |  |  |  |  |  |  |  |
| 7. Mental illness is usually a consequence of bad parenting or poor family environment |  |  |  |  |  |  |  |
| 8. People who are mentally ill do not get better |  |  |  |  |  |  |  |


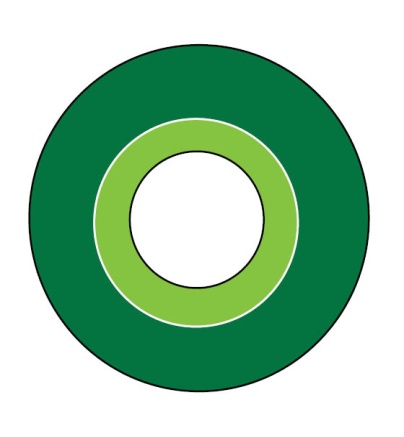

Supplement: Additional file 1: — Student Mental Health Literacy Survey. [file 12888_2014_379_MOESM1_ESM.docx]
